# Oxidative Stress and Antioxidant Therapy in Cardiovascular Diseases—Clinical Challenge

**DOI:** 10.3390/jcm11133784

**Published:** 2022-06-30

**Authors:** George Pavlidis

**Affiliations:** 2nd Department of Cardiology, Attikon University Hospital, Medical School, National and Kapodistrian University of Athens, 12462 Athens, Greece; geo_pavlidis@yahoo.gr

Oxidative stress reflects an imbalance between the production of reactive oxygen species (ROS) and the biological systems’ antioxidant mechanisms. Overproduction of ROS, such as superoxide anion (O_2_^•−^), hydroxyl ion (OH^•^) and hydrogen peroxide (H_2_O_2_), has a detrimental effect on cellular and molecular functions by damaging DNA, proteins, and lipids and by effects on redox-sensitive cellular signaling pathways [1]. In addition, an elevated production of ROS and the subsequent reduction in vascular bioavailability of nitric oxide result in endothelial dysfunction, which is a characteristic feature of atherosclerotic disease [2]. Interestingly, in the current Special Issue, Ikonomidis et al. demonstrated that decreased nitric oxide bioavailability during postprandial hyperglycemia is linked to impaired endothelial, coronary and left ventricular myocardial function in dysglycaemic subjects and first-degree relatives of diabetic patients. Authors suggested that prolonged and repeated hyperglycaemia may play an important role in the development of atherosclerosis [3]. In fact, traditional cardiovascular risk factors, namely hypertension, hyperlipidemia, diabetes mellitus, metabolic syndrome and chronic smoking, are characterized by increased oxidative stress burden [4]. Accumulating evidence show that oxidative stress plays a pivotal role in the development of various cardiovascular diseases (CVDs), which include myocardial ischemia-reperfusion injury, cardiac remodeling, atrial fibrillation, diabetic cardiomyopathy, heart failure, and chemotherapy-induced cardiotoxicity [5].

According to several clinical studies and trials, antioxidant therapies in oxidative stress-associated CVDs include four different strategies in the clinical settings: (a) inhibition of oxidative stress production through xanthine oxidase and nitric oxide synthase inhibitors, (b) enhancement of endogenous antioxidant capacity by N-acetyl cysteine administration, (c) enhancement of antioxidant capacity by administration of exogenous antioxidant supplements, and (d) administration of medications with indirect antioxidant and anti-inflammatory properties such as statins, proprotein convertase subtilisin/kexin type 9 (PCSK9) inhibitors, metformin, carvedilol and melatonin [5,6].

However, antioxidants in the prevention and treatment of CVDs remains a matter of debate [4]. Several exogenous antioxidants, like vitamin C, vitamin E, carotenoids, polyphenols, minerals, omega-3 poly-unsaturated fatty acids and olive oils, have demonstrated preventive and therapeutic benefits in the management of CVDs [5]. Recently, coenzyme Q10 has been used in clinical practice as an intensifier of mitochondrial energy metabolism and an antioxidant agent with the aim of reducing oxidative damage in a wide range of diseases including type 2 diabetes mellitus and CVDs [7]. Despite these promising research findings, poor bioavailability and variable pharmacokinetic properties of several antioxidants limit their therapeutic efficacy [8]. 

On the other hand, oxidative stress biomarkers, such as malondiadehyde and protein carbonyls, are increased in chronic inflammatory diseases and are associated with impaired vascular, coronary microcirculatory and left ventricular myocardial function [9,10]. However, in the last decade, the administration of biologic agents, namely anti-interleukin (IL)-1A (anakinra) and anti-IL-6 (tocilizumab) in rheumatoid arthritis, as well as anti-IL-12/23 (ustekinumab) and anti-IL-17A (sekukinumab) in psoriatic disease, resulted in the improvement of vascular, coronary and myocardial function in parallel with a concomitant reduction of oxidative stress. These research data suggest that the beneficial effects of biologic agents on overall cardiovascular function are partly mediated by a reduction in oxidative stress burden [9,10,11]. 

Moreover, newer antidiabetic drugs seem to have important antioxidant and cardioprotective effects [12,13]. Recent study showed that a 12-month treatment with glucagon-like peptide-1 receptor agonists (GLP-1RA), sodium-glucose cotransporter-2 inhibitors (SGLT-2i), and their combination resulted in reduction of oxidative stress, as assessed by serum levels of thiobarbituric acid reactive substances (TBARS) and malondiadehyde, and in increase of antioxidant biomarkers through estimating the serum levels of 2,2′-azino-bis-(3-ethylbenzothiazoline-6-sulfonic acid) radical (ABTS). Of particular interest, the combination of GLP-1RA and SGLT-2i was superior and additive to each separate medication and the beneficial effects appeared earlier [13]. It is worth mentioning that SGLT-2i, which are now used in non-diabetic patients with heart failure, have been shown to ameliorate cardiac oxidative stress in animal models, independently from their glucose lowering effect [14]. Experimental evidence indicates that the antioxidant and anti-inflammatory actions of SGLT-2i improve endothelial function, a key determinant of future cardiovascular events [15]. 

Novel experimental antioxidant therapies are a very promising source of agents for future clinical application. MicroRNAs (miRNAs) are endogenous non-coding RNAs molecules that regulate target genes by inhibiting protein expression or promoting messenger RNA degradation. Oxidative stress affects expression of multiple miRNAs and, conversely, miRNAs regulate many genes involved in the response of biological systems in oxidative stress [16]. Up to the present moment, several miRNAs, such as miRNA-199, miRNA-210, miRNA-144, miRNA-1, miRNA-133 and miRNA-21, have been shown to be potential biomarkers of oxidative stress-associated CVDs, including myocardial infarction, ischemia-reperfusion injury, cardiac hypertrophy, endothelial dysfunction, and heart failure [5,17]. New strategies based on modifying the expression of miRNAs, may lead to the development of a useful therapeutic option. At the same time, nanomedicine has developed a wide variety of nanomaterials, namely liposomes and nanoparticles, with unique ROS-regulating properties aiming to the emergence of a new generation therapeutic strategy [8,18]. Although promising, further trials are needed to investigate their effectiveness in the therapeutic management of CVDs.

Taking into consideration the above, the aim of the current Special Issue on “Oxidative Stress and Antioxidant Therapy in Cardiovascular Diseases—Clinical Challenge” is to focus on the major aspects of the pathophysiological mechanisms of oxidative stress and to highlight promising therapeutic options for the management of patients with oxidative stress-associated CVDs. The deeper understanding of the role of oxidative stress in the pathogenesis and progression of CVDs and a thorough analysis of known and novel antioxidant therapies will contribute substantially to the more effective primary and secondary cardiovascular prevention.
